# Effect of in-hospital evolocumab therapy on lipoprotein(a) in patients with acute myocardial infarction: a retrospective cohort study and a propensity score matching analysis

**DOI:** 10.1097/CP9.0000000000000036

**Published:** 2023-04-27

**Authors:** Ge Gao, Tao Zheng, Beidi Lan, Weiying Hui, Shi Chen, Zuyi Yuan, Yue Wu, John Y. L. Chiang, Tao Chen

**Affiliations:** 1 Department of Cardiology, the First Affiliated Hospital of Xi’an Jiaotong University, Xi’an 710061, China.; 2 Key Laboratory of Environment and Genes Related to Diseases, Ministry of Education, Xi’an Jiaotong University, Xi’an 710061, China.; 3 Integrative Medical Sciences, Northeast Ohio Medical University, Rootstown, OH 44272, USA.

**Keywords:** Acute myocardial infarction, Evolocumab, Lipoprotein(a)

## Abstract

Elevated lipoprotein(a) is associated with an increased risk of atherosclerotic cardiovascular disease. Evolocumab, a proprotein convertase subtilisin/kexin type 9 inhibitor, has been shown to reduce lipoprotein(a). However, the effect of evolocumab on lipoprotein(a) in patients with acute myocardial infarction (AMI) is poorly studied. This study aims to investigate the change in lipoprotein(a) under evolocumab therapy in patients with AMI.

**Methods:**

This retrospective cohort analysis included a total of 467 AMI patients with LDL-C level >2.6 mmol/L upon admission, among whom 132 received in-hospital evolocumab (140 mg every 2 weeks) plus statin (20 mg atorvastatin or 10 mg rosuvastatin per day) and the remaining 335 received statin only. Lipid profiles at 1-month follow-up were compared between the two groups. A propensity score matching analysis was also conducted based on age, sex, and baseline lipoprotein(a) at a 1:1 ratio using a 0.02 caliper.

**Results:**

At the 1-month follow-up, the lipoprotein(a) level decreased from 27.0 (17.5, 50.6) mg/dL to 20.9 (9.4, 52.5) mg/dL in evolocumab plus statin group, but increased from 24.5 (13.2, 41.1) mg/dL to 27.9 (14.8, 58.6) mg/dL in statin only group. The propensity score matching analysis included 262 patients (131 in each group). In subgroup analysis of the propensity score matching cohort stratified by the baseline lipoprotein(a) at cutoff values of 20 and 50 mg/dL, the absolute change in lipoprotein(a) was −4.9 (−8.5, −1.3), −5.0 (−13.9, 1.9), −0.2 (−9.9, 16.9) mg/dL in three subgroups in evolocumab plus statin group, and 0.9 (−1.7, 5.5), 10.7 (4.6, 21.9), 12.2 (2.9, 35.6) mg/dL in three subgroups in statin only group. In comparison to statin only group, evolocumab plus statin group had lower lipoprotein(a) level at 1 month in all subgroups (*P* < 0.05).

**Conclusions:**

In-hospital initiation of evolocumab on a background statin therapy reduced lipoprotein(a) level at 1-month follow-up in patients with AMI. Evolocumab plus statin therapy inhibited the increase in lipoprotein(a) in statin only therapy, regardless of the baseline lipoprotein(a) level.

## INTRODUCTION

Acute myocardial infarction (AMI) is a severe ischemic event in atherosclerotic cardiovascular disease (ASCVD), with high morbidity and mortality^[1]^. Lowering low-density lipoprotein cholesterol (LDL-C) is a critical part of the lipid management to reduce ASCVD risk^[2]^, and statins have been the cornerstone of such treatment. However, significant residual ASCVD risk remains in a proportion of patients who have achieved LDL-C target through statin therapy^[3,4]^. Non-LDL-C parameters such as lipoprotein(a), triglycerides, and triglyceride-rich lipoproteins are associated with the residual risk^[5,6]^.

Lipoprotein(a) is a cholesterol-laden LDL-like particle composed of apolipoprotein(a) and apolipoprotein B-100. Lipoprotein(a) concentration is mainly determined by the *LPA* gene and displays large interindividual variation that could range from <1 to >200 mg/dL^[7,8]^. The distribution of lipoprotein(a) concentration is highly skewed, with >50 mg/dL in approximately 20% of the people. Genetic and epidemiological studies have demonstrated that *LPA* genotype is significantly associated with cardiovascular diseases^[9,10]^. High lipoprotein(a) level has also been confirmed to be a strong risk of ASCVD independent of other conventional risk factors^[11]^. Therapies that lower lipoprotein(a), thus, may have an additional benefit in reducing ACSVD risk.

Proprotein convertase subtilisin/kexin type 9 (PCSK9) is an enzyme that regulates serum LDL-C by degrading the LDL receptor. PCSK9 inhibition promotes LDL-receptor-mediated clearance of serum LDL-C. PCSK9 inhibitors (eg, evolocumab) have emerged as a new class of lipid-lowing drugs with a rapid LDL-C lowing effect^[12–16]^. In randomized controlled trials (RCTs) in patients with hypercholesterolemia and stable cardiovascular disease, evolocumab reduced LDL-C as well as lipoprotein(a)^[17]^. Reduction in LDL-C and lipoprotein(a) has also been shown with alirocumab, another PCSK9 antibody^[18]^. Most notably, statins had been shown to increase lipoprotein(a) in some albeit not all studies^[19–21]^.

We conducted a retrospective study to examine the potential effects of in-hospital evolocumab therapy on lipoprotein(a) among AMI patients. Results are reported below.

## METHODS

### Study population and design

This was a single-center, retrospective, observational cohort study. We screened all patients admitted for AMI during a period from January 2019 to October 2022 at the Department of Cardiology, the First Affiliated Hospital of Xi’an Jiaotong University. The diagnosis of AMI was based on the American Cardiology College Guidelines^[22]^. LDL-C level must be >2.6 mmol/L upon admission for inclusion in the analysis since the current guidelines recommended evolocumab for intensive lipid-lowering therapy^[23]^. Patients receiving PCSK9 inhibitor treatment before admission and those who died during hospitalization were excluded from the analysis. Evolocumab (140 mg every 2 weeks) was initiated in hospital according to the Chinese consensus on dyslipidemia management in patients with very high-risk ASCVD^[23]^. Statin therapy (20 mg atorvastatin or 10 mg rosuvastatin per day) was based on the current AMI guidelines. Data were extracted from the Biobank of the First Affiliated Hospital of Xi’an Jiaotong University. Follow-up of blood lipid profile was performed approximately 1 month after discharge. The study conformed to the provisions of the *Declaration of Helsinki* (as revised in 2013) and was approved by the Ethics Committee of the First Affiliated Hospital of Xi’an Jiaotong University (XJTU1AF20220LSL-007; date: November 13, 2020). Due to the retrospective nature of the study, informed consent was waived by the Ethics Committee.

### Propensity score match

A propensity score matching analysis was conducted to better evaluate the effect of evolocumab. Matched variables included age, sex, and baseline lipoprotein(a) level. Propensity score matching was performed using the nearest-neighbor 1:1 matching method with a match tolerance of 0.02.

### Statistical analysis

Continuous variables following normal distribution were analyzed using Student *t* test and expressed as mean ± standard deviation. Continuous variables not following normal distribution were analyzed using Mann-Whitney *U* test and expressed as median and quartiles (quartile 1 [Q1], quartile 3 [Q3]). Normality was assessed by the Kolmogorov-Smirnov test. Pre- and after- comparison was conducted using Student *t* test for paired data or Wilcoxon test, as appropriate. Categorical variables were analyzed using Chi-square or the Fisher test as appropriate and represented as a percentage. All statistical analyses were conducted using SPSS v27 for Windows (IBM Corp., Armonk, NY, USA) and GraphPad 9.0 Prism (GraphPad Software, San Diego, CA, USA). The difference was considered statistically significant at *P* < 0.05.

## RESULTS

### Baseline characteristics

The final analysis included 467 AMI patients, among which 132 patients received evolocumab plus statin the rapy and 335 patients received statin-only therapy. The median age was 57.0 (49.0, 67.0) years, and 79.0% of patients were men. At the baseline, only 13 patients were receiving any statin therapy (Table 1). In comparison to the statin only group, patients in evolocumab plus statin group had a higher level of LDL-C, lipoprotein(a), cholesterol, triglyceride, apolipoprotein B, and apolipoprotein E, and a lower level of apolipoprotein A. The median LDL level was 3.5 (3.2, 4.1) mmol/L in evolocumab plus statin group and 3.1 (2.8, 3.4) mmol/L in statin only group (*P* < 0.001). The median lipoprotein(a) level was 27.0 (17.5, 50.6) and 24.5 (13.2, 41.1) mg/dL, respectively (*P* = 0.038).

**Table 1.** Demographic and baseline characteristics

	Evolocumab + statin (*n* = 132)	Statin (*n* = 335)	*P* value
Age, y	56.0 (49.0, 63.0)	58.0 (49.0, 67.0)	0.075
Male, *n* (%)	106 (80.3)	263 (78.5)	0.668
Hypertension, *n* (%)	51 (38.6)	154 (46.0)	0.150
Diabetes mellitus, *n* (%)	20 (15.2)	75 (22.4)	0.08
Statin use	6 (4.5)	7 (2.1)	0.254
eGFR, mL/min/1.73 m^2^	107.8 (94.2, 119.6)	107.7 (96.2, 118.7)	0.584
CRP, mg/L	4.3 (2.3, 16.2)	4.4 (1.4, 21.1)	0.791
Cholesterol, mmol/L	5.4 (4.8, 6.1)	5.0 (4.6, 5.6)	<0.001
Triglyceride, mmol/L	1.7 (1.3, 2.4)	1.5 (1.1, 2.0)	0.002
HDL-C, mmol/L	1.0 (0.9, 1.1)	1.0 (0.9, 1.1)	0.202
Apolipoprotein A, g/L	1.0 (0.9, 1.2)	1.1 (1.0, 1.2)	0.002
Apolipoprotein B, g/L	1.1 (1.0, 1.2)	1.0 (0.9, 1.1)	<0.001
Apolipoprotein E, mg/L	42.3 (35.1, 53.2)	40.0 (33.2, 49.3)	0.039
LDL-C, mmol/L	3.5 (3.2, 4.1)	3.1 (2.8, 3.4)	<0.001
Lipoprotein(a), mg/dL	27.0 (17.5, 50.6)	24.5 (13.2, 41.1)	0.038

Data are shown as median (Q1, Q3).

CRP: C-reactive protein; eGFR: Estimated glomerular filtration rate; HDL-C: High-density lipoprotein cholesterol; LDL-C: Low-density lipoprotein cholesterol.

### Lipid profile at 1-month follow-up

**Figure 1.**
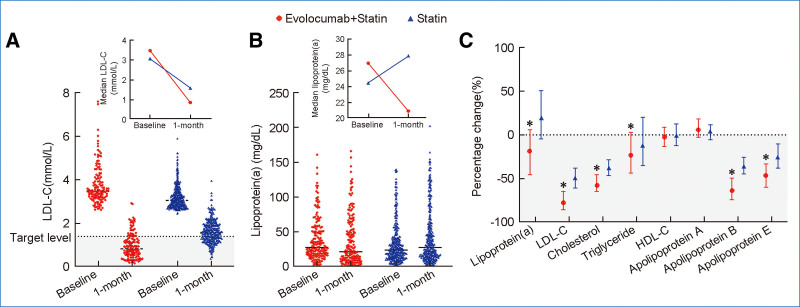
**Lipid profile at the baseline and 1-month follow-up in the overall cohort. A**, Distribution and changes in LDL-C from baseline to 1-month follow-up. Target LDL-C level was 1.4 mmol/L. **B**, Distribution and changes in lipoprotein(a) from baseline to 1-month follow-up. **C**, Percent change in lipid profile from baseline to 1-month follow-up. Data are shown as median (Q1, Q3). *P < 0.05 versus statin group. HDL-C: high-density lipoprotein cholesterol; LDL-C: low-density lipoprotein cholesterol.

In comparison to the baseline, the level of LDL-C, cholesterol, triglyceride, apolipoprotein B, and apolipoprotein E at 1 month was lower in both groups (Table 2 and Figure 1), but the magnitude of reduction was more robust in the evolocumab plus statin group (*P* < 0.05 for all comparisons). The absolute LDL-C reduction was −2.8 mmol/L (from a median of 3.5 mmol/L to 0.8 mmol/L, *P* < 0.001) in the evolocumab plus statin group versus −1.6 mmol/L (from a median of 3.1 mmol/L to 1.6 mmol/L, *P* < 0.001) in the statin only group (*P* < 0.001). Percent change relative to the baseline was −77.9% versus 49.2% in the two groups (*P* < 0.001). LDL-C was reduced to the target goal of < 1.4 mmol/L in 77.2% in the evolocumab plus statin group versus 35.5% in the statin only group, respectively (*P* < 0.001).

**Table 2.** Change in lipid profile at 1-month follow-up in the overall cohort

	Evolocumab + statin (*n* = 132)	Statin (*n* = 335)	*P* value
Lipoprotein(a)			
Baseline, mg/dL	27.0 (17.5, 50.6)	24.5 (13.2, 41.1)	0.038
1-month, mg/dL	20.9 (9.4, 52.5)	27.9 (14.8, 58.6)	0.018
Absolute change, mg/dL	−4.7 (−9.9, 1.3)	5.1 (−1.0, 14.1)	<0.001
Percent change, %	−18.6 (−46.0, 5.8)	19.8 (−4.5, 50.5)	<0.001
LDL-C			
Baseline, mmol/L	3.5 (3.2, 4.1)	3.1 (2.8, 3.4)	<0.001
1-month, mmol/L	0.8 (0.5, 1.3)	1.6 (1.3, 1.9)	<0.001
Absolute change, mmol/L	−2.8 (−3.2, −2.3)	−1.6 (−2.0, −1.1)	<0.001
Percent change, %	−77.9 (−86.1, −64.6)	−49.2 (−60.7, −38.5)	<0.001
Under target at 1 month, *n* (%)	102 (77.2)	119 (35.5)	<0.001
Percent change for other lipids, %			
Cholesterol	−58.0 (−64.9, −46.1)	−38.2 (−46.8, −28.9)	<0.001
Triglyceride	−23.3 (−44.1, 2.7)	−11.9 (−35.3, 20.3)	0.004
HDL-C	−2.5 (−13.4, 9.2)	−0.8 (−12.6, 12.9)	0.288
Apolipoprotein A	5.8 (−3.2, 18.1)	4.4 (−5.9, 11.7)	0.079
Apolipoprotein B	−64.0 (−74.3, −49.8)	−36.0 (−44.8, −26.0)	<0.001
Apolipoprotein E	−46.7 (−59.8, −33.3)	−25.5 (−38.3, −9.9)	<0.001

Data are shown as median (Q1, Q3).

HDL-C: High-density lipoprotein cholesterol; LDL-C: Low-density lipoprotein cholesterol.

Lipoprotein(a) alterations varied among two groups (Figure 1B). In comparison to the baseline, lipoprotein(a) level at 1-month follow-up was reduced in evolocumab plus statin group (from a median of 27.0 mg/dL to 20.9 mg/dL, *P* < 0.001) but increased in statin only group (from a median of 24.5 mg/dL to 27.9 mg/dL, *P* < 0.001). The absolute change was −4.7 (−9.9, 1.3) mg/dL in evolocumab plus statin group versus 5.1 (−1.0, 14.1) mg/dL in statin only group (*P* < 0.001); percent change was −18.6% (−46.0%, 5.8%) versus 19.8% (−4.5%, 50.5%) (*P* < 0.001). In comparison to the statin only group, patients in evolocumab plus statin group had lower lipoprotein(a) at 1-month follow-up (*P* = 0.018).

### Propensity score matching analysis

The propensity score matching analysis included 262 patients (131 in each group). The baseline lipoprotein(a) level was well-balanced as shown in Table 3. In the 1-month follow-up, the median lipoprotein(a) level was reduced to 20.8 (9.4, 50.6) mg/dL from baseline 26.1 (17.5, 50.4) mg/dL (*P* < 0.001) in evolocumab plus statin group, but increased to 30.7 (14.2, 63.5) mg/dL from baseline 24.7 (13.1, 45.2) mg/dL (*P* < 0.001) in statin only group. The absolute change was −4.7 (−9.9, 1.4) mg/dL versus 5.4 (−0.1, 14.7) mg/dL (*P* < 0.001), and the percent change was −18.6% (−46.0%, 5.8%) versus 20.8% (−1.1%, 52.2%), respectively (*P* < 0.001).

**Table 3.** Change in lipoprotein(a) stratified by baseline lipoprotein(a) level at 1-month follow-up in propensity-score matching analysis.

	All Matched Population			Lipoprotein(a) <20 mg/dL		
	Evolocumab + Statin	Statin	*P* value	Evolocumab + Statin	Statin	*P* value
Number of patients	131	131		44	59	
Baseline, mg/dL	26.1 (17.5, 50.4)	24.7 (13.1, 45.2)	0.141	15.0 (10.4, 17.5)	12.7 (10.2, 15.9)	0.16
1-month, mg/dL	20.8 (9.4, 50.6)	30.7 (14.2, 63.5)	0.025	7.4 (3.9, 12.2)	13.6 (8.3, 19.7)	<0.001
Absolute change, mg/dL	−4.7 (−9.9, 1.4)	5.4 (−0.1, 14.7)	<0.001	−4.9 (−8.5, −1.3)	0.9 (−1.7, 5.5)	<0.001
Percent change, %	−18.6 (−46.0, 5.8)	20.8 (−1.1, 52.2)	<0.001	−39.3 (−60.7, −19.6)	9.9 (−16.0, 50.0)	<0.001
	**20≤ Lipoprotein(a) <50 mg/dL**			**Lipoprotein(a) ≥50 mg/dL**		
	**Evolocumab + Statin**	**Statin**	***P* value**	**Evolocumab + Statin**	**Statin**	***P* value**
Number of patients	52	41		35	31	
Baseline, mg/dL	30.7 (23.8, 37.3)	31.0 (25.6, 39.2)	0.462	70.7 (56.5, 93.3)	79.3 (61.6, 110.5)	0.169
1-month, mg/dL	21.9 (15.4, 41.1)	42.2 (32.2, 55.8)	<0.001	75.8 (50.6, 112.8)	109.0 (63.5, 128.6)	0.014
Absolute change, mg/dL	−5.0 (−13.9, 1.9)	10.7 (4.6, 21.9)	<0.001	−0.2 (−9.9, 16.9)	12.2 (2.9, 35.6)	0.005
Percent change, %	−18.6 (−44.5, 7.5)	41.9 (13.3, 63.7)	<0.001	−0.2 (−16.0, 17.6)	20.0 (3.8, 47.4)	0.004

Data are shown as median (Q1, Q3). The matching was based on age, sex, and baseline lipoprotein(a) level.

**Figure 2.**
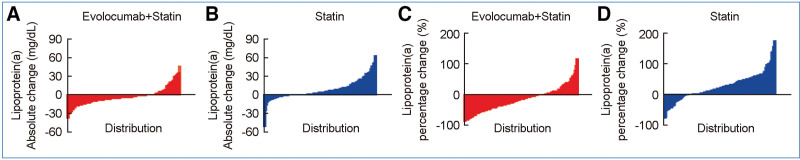
**Waterfall plot of change in lipoprotein(a) from baseline to 1-month follow-up in propensity score matching population. A**, Absolute change in evolocumab plus statin group. **B**, Absolute change in statin group. **C**, Percentage change in evolocumab plus statin group. **D**, Percentage change in statin group. The matching was based on age, sex, and baseline lipoprotein(a) level.

The range of absolute and percent change in lipoprotein(a) in the two groups is shown in Figure 2. The plot demonstrated significant individual variability. The absolute change ranged from −38.1 mg/dL to +48.2 mg/dL in evolocumab plus statin group, and from −51.0 mg/dL to +64.8 mg/dL in statin only group. The percent change ranged from −90.0% to +110.1% in evolocumab plus statin group, and from −78.1% to +173.9% in statin only group. The majority of the patients in evolocumab plus statin group (70.2%) had a reduction in lipoprotein(a), whereas the majority of the patients in statin only group (74.0%) had an increase in lipoprotein(a).

### Analysis stratified by baseline lipoprotein(a) level

**Figure 3.**
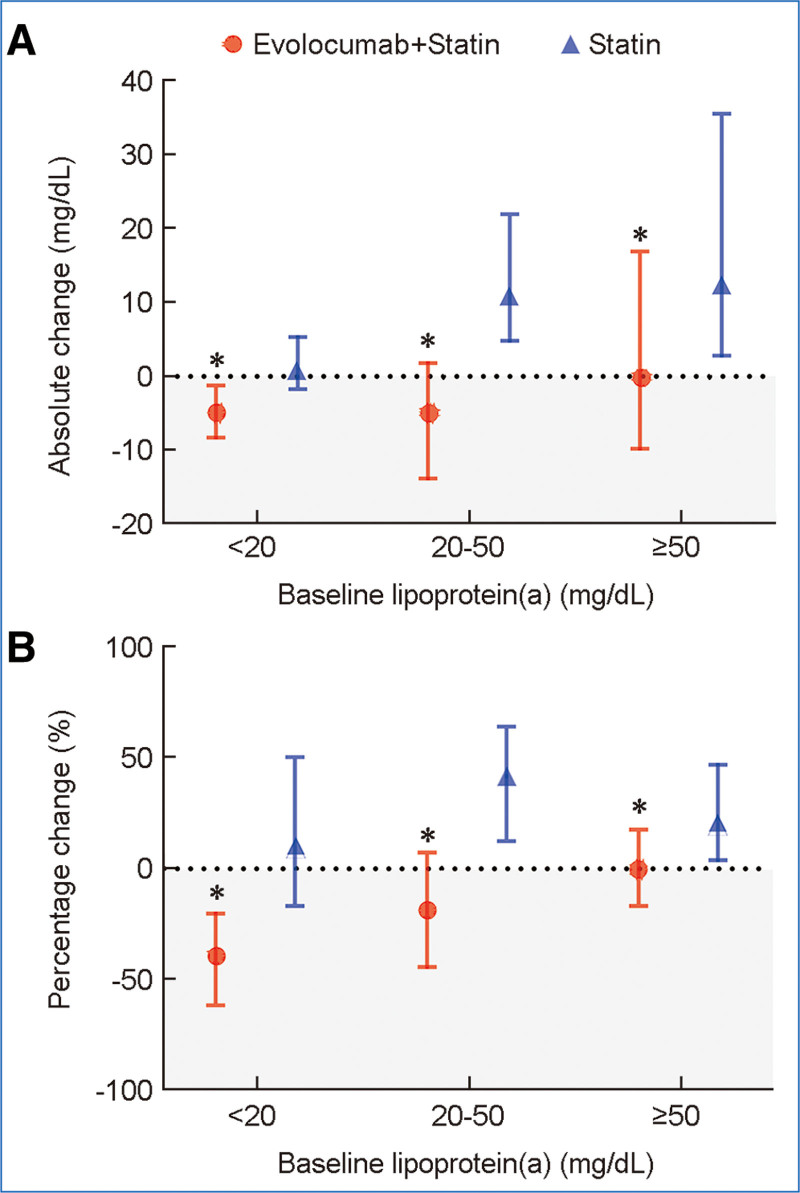
**Change in lipoprotein (a) stratified by baseline lipoprotein(a) in propensity score matching analysis. A**, Absolute change from baseline to 1 month. **B**, Percent change from baseline to 1 month. Data are shown as median (Q1, Q3). *P < 0.05 versus statin group. The matching was based on age, sex, and baseline lipoprotein(a) level.

The propensity score matching cohort was stratified into three subgroups based on the baseline lipoprotein(a) level (20 and 50 mg/dL as the cutoff values) for further analysis (Table 3 and Figure 3). In the analysis that only included patients with baseline lipoprotein(a) < 20 mg/dL, the absolute and percent change of lipoprotein(a) in evolocumab plus statin group at 1-month follow-up was −4.9 (−8.5, −1.3) mg/dL and −39.3% (−60.7%, −19.6%), respectively, with median reduction from 15.0 to 7.4 mg/dL (*P* < 0.001). In the analysis that only included patients with 20 ≤ baseline lipoprotein(a) < 50 mg/dL, the absolute and percent change of lipoprotein(a) in evolocumab plus statin group was −5.0 (−13.9, 1.9) mg/dL and −18.6% (−44.5%, 7.5%), respectively, with median reduction from 30.7 to 21.9 mg/dL (*P* < 0.001). In the analysis that only included patients with baseline lipoprotein(a) ≥ 50 mg/dL, there was no statistically significant reduction in lipoprotein(a) in the evolocumab plus statin group at 1 month (from a median of 70.7 mg/dL to 75.8 mg/dL, *P* = 0.745). In statin only group, lipoprotein(a) increased significantly at 1-month follow-up in all subgroups: the percent change was 9.9% in patients with baseline lipoprotein(a) < 20 mg/dL (from a median of 12.7 mg/dL to 13.6 mg/dL, *P* = 0.019), 41.9% in patients with 20 ≤ baseline lipoprotein(a) < 50 mg/dL (from a median of 31.0 mg/dL to 42.2 mg/dL, *P* < 0.001) and 20.0% in patients with baseline lipoprotein(a) ≥ 50 mg/dL (from a median of 79.3 mg/dL to 109.0 mg/dL, *P* < 0.001). The lipoprotein(a) level at 1 month was lower in evolocumab plus statin group than in statin only group in all subgroups (*P* < 0.05 for all comparisons).

## DISCUSSION

The results of the current study showed in-hospital initiation of evolocumab therapy on a background statin therapy significantly reduced lipoprotein(a) level at 1-month follow-up in patients with AMI. The lipoprotein(a) level was decreased by a median of 18.6% in evolocumab plus statin group but increased by a median of 19.8% in statin only group. No significant change in lipoprotein(a) level was observed in evolocumab plus statin group with a baseline lipoprotein(a) ≥ 50 mg/dL. In comparison to statin only group, lipoprotein(a) level at 1 month was significantly lower in evolocumab plus statin group regardless of baseline lipoprotein(a).

A meta-analysis of RCTs^[17]^ that compared either evolocumab or alirocumab with placebo and/or other lipid-lowering drugs showed a 26.7% reduction in lipoprotein(a) (28.17% for evolocumab and 24.53% for alirocumab). But among the 41 trials included in the meta-analysis, only one study (EVOPACS) was performed in patients with the acute coronary syndrome. In the EVOPACS study^[16]^, there was a significant absolute reduction in lipoprotein(a) at 8 weeks after treatment with evolocumab plus statin, but no significant percent reduction relative to baseline. An RCT of 102 Japanese AMI patients^[24]^ reported a percent change of −2.7% ± 48.6% in lipoprotein(a) at 4 weeks after treatment with evolocumab plus statin. Evolocumab plus statin did not significantly lower lipoprotein(a) versus the baseline but seemed to attenuate the lipoprotein(a) increase in statin therapy. We observed similar results in the subgroup analysis that included only patients with baseline lipoprotein(a) ≥ 50 mg/dL. These results suggest evolocumab could inhibit the increase of lipoprotein(a) in statin therapy.

The results of subgroup analysis confirmed lipoprotein(a)-lowering effect of evolocumab plus statin therapy in patients with baseline lipoprotein(a) below 50 mg/dL, as reflected by both absolute and percent change after the treatment. Effects in patients with baseline lipoprotein(a) ≥ 50 mg/dL seemed less robust. In the Further Cardiovascular Outcomes Research with PCSK9 Inhibition in Subjects with Elevated Risk (FOURIER) trial^[25]^, the absolute reduction in lipoprotein(a) upon evolocumab treatment was greatest in individuals with higher baseline lipoprotein(a) level, but the percent reduction decreased with increasing baseline lipoprotein(a) level. In the ODYSSEY Outcomes trial^[18]^, the median absolute reduction upon alirocumab treatment also decreased with increasing baseline lipoprotein(a) level.

In the current study, lipoprotein(a) level at the 1-month follow-up in statin only group was significantly higher than the baseline, regardless of the baseline lipoprotein(a) level. The results are consistent with a recent meta-analysis^[19]^, in which statin therapy significantly increased lipoprotein(a) level from baseline. Another meta-analysis in 2022^[20]^, however, failed to show clinically important differences in lipoprotein(a) in patients undergoing statin treatment versus placebo. Whether the observed elevation of lipoprotein(a) level in the statin groups is directly caused by statin treatment is not clear. It is also likely that increased lipoprotein(a) level merely represents a response to increase proinflammatory cytokines in the acute phase of AMI^[26]^.

Lipoprotein(a) is an independent risk factor of ASCVD. A mendelian randomization analysis concluded that a lipoprotein(a) reduction of 65.7 mg/dL is required to achieve a similar cardiovascular benefit as that produced by the 38.7 mg/dL reduction of LDL-C^[27]^. Recent clinical trials provided further support for the relationship between lipoprotein(a) concentration and ASCVD. Results from the FOURIER trial demonstrated an association between elevated baseline lipoprotein(a) level with increased risk of major coronary events even after multivariable adjustment, and evolocumab tended to reduce the risk of major coronary events to a greater degree in patients with higher baseline lipoprotein(a) level^[25]^. ODYSSEY Outcomes study suggested a linear relationship between baseline lipoprotein(a) level and the risk of major adverse cardiovascular events, adding further support to the notion that reducing lipoprotein(a) level with alirocumab could decrease cardiovascular risk^[18]^. Decreased lipoprotein(a) level in AMI patients receiving evolocumab plus statin therapy in the current study suggested additional benefits with evolocumab in AMI patients.

In comparison to statin-only therapy, our results showed that evolocumab plus statin therapy was more effective in lowering lipids at 1-month follow-up. The percentage of patients who achieved LDL-C target level at 1 month in evolocumab plus statin group was twice higher than in statin only group. The magnitude of reduction in cholesterol, triglyceride, apolipoprotein B, and apolipoprotein E was also greater than statin only group.

The current study has several limitations. First, the single-center, retrospective, observational design introduces a variety of biases. Second, data were available only at the 1-month follow-up. The relatively small sample size also limits the validity of subgroup analysis. Prospective studies of a larger sample size and long-term follow-up are needed.

## CONCLUSIONS

In conclusion, in-hospital initiation of evolocumab on a background statin therapy reduced lipoprotein(a) level at 1-month follow-up in patients with AMI. Evolocumab plus statin therapy inhibited the increase in lipoprotein(a) in statin only therapy regardless of the baseline lipoprotein(a) level.

## FUNDING

This work was supported by the National Key R&D Program of China (2019YFA0802300 to YW), the National Natural Science Foundation of China (81970351 to YW, 81870330 to TC), National Institutes of Health grants (DK44442 and DK58379 to JYLC), and the Clinical Research Award of the First Affiliated Hospital of Xi’an Jiaotong University, China (XJTU1AF-CRF-2020-007 to TC).

## AUTHOR CONTRIBUTIONS

TC, YW, and JYLC participated in research design; BDL, WYH, and SC contributed to data collection; GG and TZ participated in data analysis; GG participated in manuscript writing. All authors reviewed and approved the manuscript.

## CONFLICTS OF INTEREST STATEMENT

The authors declare that they have no conflict of interest with regard to the content of this manuscript.

## ACKNOWLEDGMENT

We thank Dr. Lu Ma from the School of Public Health, Global Health Institute, Xi’an Jiaotong University Health Science Center for providing statistical consultation, and the Biobank of First Affiliated Hospital of Xi’an Jiaotong University for providing clinical data.

## DATA SHARING STATEMENT

Research data are available upon request to the corresponding author.
